# Five-year comparative outcomes of the track technique versus conventional artificial chordae sizing in anterior mitral leaflet repair

**DOI:** 10.3389/fcvm.2025.1642204

**Published:** 2025-09-09

**Authors:** Giuseppe Nasso, Walter Vignaroli, Raffaele Bonifazi, Giovanni Valenti, Flavio Fiore, Dritan Hila, Tommaso Loizzo, Rosalba Franchino, Antongiulio Valenzano, Giacomo Errico, Vincenza Vitobello, Giuseppe Balducci, Giacomo Schinco, Felice Agrò, Mario Siro Brigiani, Cataldo Girasoli, Guido Lembo, Ernesto Greco, Gaetano Contegiacomo, Giuseppe Santarpino, Giuseppe Speziale

**Affiliations:** ^1^ Department of Cardiac Surgery, Anthea Hospital and Santa Maria Hospital GVM Care&Research, Bari, Italy; ^2^ Department of Cardiac Surgery, San Carlo di Nancy Hospital GVM Care&Research, Rome, Italy; ^3^ Department of Cardiology Azienda Ospedaliera B.A.T., Bonomo Hospital, Andria, Italy; ^4^ Anesthesia and Intensive Care Research Unit, Campus Bio-Medico University, Rome, Italy; ^5^ Department of Health and Life Sciences European, University of Rome, Rome, Italy; ^6^ Department of Cardiac Surgery, Città di Lecce Hospital, GVM Care & Research, Lecce, Italy; ^7^ Department of Clinical and Experimental Medicine, Magna Graecia University, Catanzaro, Italy; ^8^ Department of Cardiac Surgery, Paracelsus Medical University, Nuremberg, Germany

**Keywords:** mitral regurgitation, anterior leaflet prolapse, artificial chordae, mitral repair, mitral valve surgery

## Abstract

**Introduction:**

Determining artificial chordae length is crucial for successful mitral valve repair (MVr). This study evaluates five-year outcomes of a novel “track technique”, which uses an annular guiding device for chordal length adjustment, compared to a conventional approach.

**Methods:**

A retrospective analysis was conducted on 47 patients who underwent MVr with artificial chordae: 25 received the track technique, and 22 underwent conventional chordal sizing. All patients received complete annuloplasty and were followed for five years. The primary endpoint was freedom from moderate or severe mitral regurgitation (MR); secondary endpoints included NYHA class, coaptation length, freedom from reintervention, and all-cause mortality.

**Results:**

At five years, neither group showed moderate/severe MR or required reoperation. However, the track group showed superior outcomes: significantly longer coaptation length (10.7 ± 1.5 mm vs. 8.6 ± 1.8 mm, *p* = 0.03) and lower residual MR (12% vs. 32%, *p* = 0.04). More than 90% of patients in both groups were in NYHA class I–II.

**Discussion:**

In conclusion, the track technique is a safe, effective, and reproducible method for artificial chordae sizing in MVr. It ensures better leaflet coaptation, reduces residual MR, and maintains favorable clinical results over a five-year period.

## Introduction

Degenerative mitral regurgitation (DMR) is a prevalent cardiac condition and represents one of the most frequent indications for mitral valve surgery in developed countries. In recent decades, mitral valve repair (MVr) has emerged as the preferred treatment strategy over valve replacement due to its superiority in preserving left ventricular function, reducing thromboembolic risk, avoiding prosthesis-related complications, and improving long-term survival. This paradigm shift has been supported by numerous studies demonstrating durable outcomes and favorable hemodynamics following repair, particularly in patients with isolated leaflet prolapse or flail (1–4).

Anterior leaflet prolapse or flail, while less common than posterior leaflet disease, presents unique surgical challenges due to the intrinsic mobility and length of the anterior leaflet and the complexity of achieving adequate coaptation. Successful repair in such cases often relies on the use of artificial chordae tendineae, typically constructed from expanded polytetrafluoroethylene (ePTFE), which provide a durable alternative to leaflet resection and preserve leaflet tissue. However, despite their advantages, a key limitation of artificial chordae is the difficulty of accurately determining the appropriate chordal length intraoperatively, which directly impacts the quality of the repair and its long-term durability (5–7).

Traditionally, chordal length estimation has depended on visual approximation, traction testing, or the saline test to simulate ventricular pressurization. These methods are subjective, operator-dependent, and may be affected by inter-patient anatomical variability and intraoperative conditions. Moreover, these approaches do not allow for real-time measurement under physiological loading conditions, leading to potential discrepancies in coaptation and leaflet tension. Inconsistent outcomes may contribute to early residual mitral regurgitation (MR), increased reintervention rates, and reduced repair durability.

To overcome these limitations, several innovations have been introduced. Techniques such as the loop technique, premeasured chords, and template-guided implantation have attempted to standardize neo-chordal length. Notably, the Chord-X system offers pre-measured loops of varying lengths to facilitate implantation and has demonstrated promising results, particularly in low-volume surgical centers and in minimally invasive settings. Similarly, the Leipzig Loop technique, the loop-in-loop method, and papillary muscle relocation have been explored. While effective, these approaches may still require experience or auxiliary instrumentation and may not be universally applicable or reproducible (8–10).

In this context, we previously introduced the “track technique” as a novel and standardized approach for artificial chordae length determination. This method utilizes a temporary annular guiding device inserted after the annuloplasty ring placement. The guide establishes a consistent reference point for chordae anchoring and eliminates the need for ventricular pressurization or subjective caliper-based measurement. Early results demonstrated that the track technique produced more uniform coaptation lengths and reduced early residual MR when compared to conventional estimation methods (11).

Although preliminary data from short-term follow-up were encouraging, long-term outcomes and durability of the track technique remained to be confirmed. This study builds upon our prior observations and presents a 5-year comparative analysis of the clinical and echocardiographic results of the track technique vs. a conventional visual estimation method in patients undergoing MVr for anterior leaflet prolapse or flail. In doing so, we aim to provide robust evidence supporting the technique's long-term efficacy, reproducibility, and suitability for broader clinical adoption.

In this setting, the need for reproducible and efficient intraoperative techniques is amplified by the rising demand for minimally invasive approaches. As more centers adopt robotic and video-assisted MVr, the ability to execute high-quality repairs with limited exposure becomes increasingly dependent on standardized tools and approaches such as the track technique (12–16).

## Materials and methods

This retrospective observational study was conducted at a single high-volume mitral center, Anthea Hospital – GVM Care & Research - and adheres to the STROBE guidelines for observational studies. The study protocol was approved by our Institutional Review Board. IRB numb. 5.2025.

All patients provided informed consent for data collection, analysis, and long-term follow-up. Pre, intra, and postoperative outcomes were compared between the 2 groups. All patients were contacted for a follow-up every year up to a maximum of 5 years. The aim of this investigation was to compare long-term outcomes between two different techniques for artificial chordae sizing during mitral valve repair (MVr) in patients with MR due to anterior leaflet prolapse.

### Study population

Between January 2020 and January 2022, 47 patients with isolated anterior mitral leaflet prolapse or flail underwent MVr with artificial chordae implantation. Inclusion criteria were: symptomatic mitral regurgitation (MR) graded at least moderate (≥2+), eligibility for valve repair without need for concomitant procedures, and deemed technically suitable for artificial chordae implantation.

Feasibility was determined pre- and intra-operatively as follows:

• Anatomy: degenerative isolated anterior leaflet prolapse (A1–A3) with sufficient leaflet tissue to obtain an adequate coaptation zone after annuloplasty (our target is ≥8 mm).
• Anchoring: ability to securely expose and fix PTFE neochordae to the corresponding papillary muscle head without undue tension.
• Exclusions (unsuitable for the technique): rheumatic/restrictive valve disease, active endocarditis, relevant leaflet/annular calcification hampering suture passage, commissural clefts/perforations requiring patch/resection, papillary-muscle pathology precluding safe anchoring, functional/ischemic tethering, or intra-operative prediction of persistent leaflet restriction/SAM despite annuloplasty on the saline test/TEE.

Exclusion criteria included: posterior or bileaflet prolapse, rheumatic valve disease, infective endocarditis, atrial fibrillation requiring surgical treatment, or prior mitral valve surgery. Patient with acute MR were included in the study. This population was then compared with a similar group of patients who underwent surgery using the traditional technique.

### Surgical procedure and techniques

**Figure 1:**
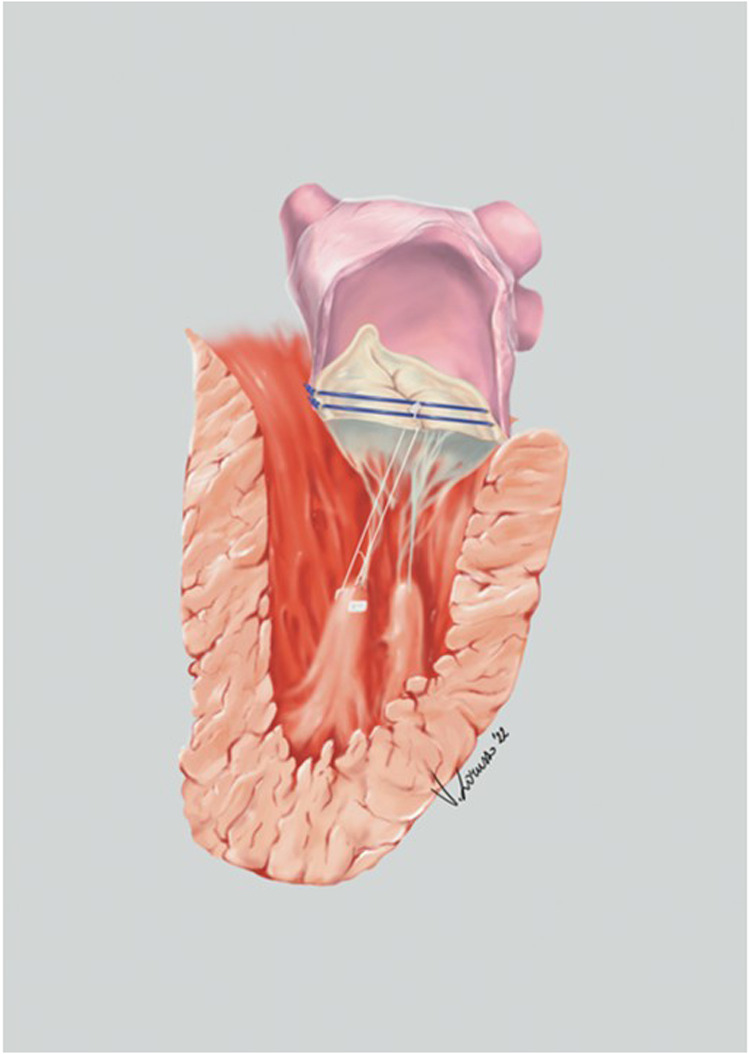
Anatomical illustration of mitral valve repair using track technique.

All procedures were performed under general anesthesia (Figure 1) with transesophageal echocardiographic guidance (performed by a certified cardiologist or anesthesiologist). Cardiopulmonary bypass was established via femoral cannulation, and myocardial protection was achieved with antegrade and/or retrograde cold blood cardioplegia. Surgical access to the mitral valve was usually achieved through a right mini-thoracotomy in the third or fourth intercostal space, depending on the patient's anatomy, in some patients was necessary a sternotomy access (Table 1).

**Table 1:** Preoperative characteristics of patients undergoing mitral valve repair.

Variable	Track group (*n* = 25)	Conventional group (*n* = 22)	*p*-value
Age (years) ± SD	72.5 ± 4.22	71.8 ± 2.11	0.47
Male sex (%)	16 (64%)	15 (68%)	0.78
NYHA class III–IV (%)	7 (28%)	7 (32%)	0.72
Left ventricular ejection fraction (%)± SD	58 ± 6	57 ± 5	0.65
Atrial fibrillation (%)	20%	18%	0.85
Isolated anterior prolapse (%)	100%	100%	—
Mini-thoracotomy access (%)	80%	77%	0.81
MR grade (%)	Severe 22%–92%	Severe (21)—95%	0.75
	Moderate 3%–8%	Moderate (1)—5%	

In this study we included only patients with isolated anterior leaflet prolapse to keep the cohort anatomically homogeneous and to assess the technique in the context where it was first standardized. The track technique, however, is not limited to anterior lesions: it is equally applicable to posterior leaflet prolapse, with the same operative steps and goal of precise neochordae length/leaflet height setting. Posterior cases were not analyzed here because they were outside the prespecified cohort, but we agree that extending the evaluation to posterior lesions is clinically relevant and plan to address this in future work.

Patients were assigned to two groups based on the intraoperative chordal sizing technique:

– Track Technique Group (*n* = 25): After implantation of a complete annuloplasty ring (MEMO 3D, LivaNova), a temporary annular guide was introduced through the left atrium to define a reference plane for chordal length. Neo-chordae made of CV-4 ePTFE (Gore-Tex) were anchored to the papillary muscle tips and secured to the prolapsing anterior leaflet segment. The guide ensured chordae were tied off at a constant height corresponding to the annular level.
– Conventional Group (*n* = 22): After implantation of a complete annuloplasty ring (MEMO 3D, LivaNova), chordal length was estimated via visual alignment with adjacent leaflet segments, native chordae, and intraoperative saline testing under direct vision. Surgeons adjusted the chordal tension manually until acceptable coaptation was achieved, according to their experience and discretion.

In both groups, chordae were implanted using pledgeted mattress sutures, typically two per prolapsing segment. Leaflet resection was not performed in any case.

Group allocation was determined intraoperatively by the chordal sizing strategy actually used:

• Track group: chordal length set with the track technique after complete annuloplasty.
• Conventional group: chordal length set by conventional visual estimation after annuloplasty.

The choice of strategy was left to the operating surgeon, based on intraoperative assessment (leaflet tissue/height, exposure of the corresponding papillary muscle head, saline test/TEE). Surgeons were experienced with both approaches and leaflet resection was not performed in either group.

Switching intraoperatively: if findings suggested that the initially intended approach would not provide optimal coaptation, the surgeon could change strategy. For analysis, patients were classified according to the final sizing method used (as-treated).

### Echocardiographic assessment

Transthoracic echocardiography (TTE) was performed preoperatively and at each follow-up visit. Transesophageal echocardiography (TEE) was used intraoperatively and at early postoperative stages to confirm repair adequacy. Standardized views were acquired: mid-esophageal long-axis and four-chamber views for coaptation assessment, and biplane views for leaflet motion and residual MR evaluation.

Coaptation length was measured intraoperatively using TEE with color Doppler mapping. Measurements were performed in systole along the central scallop (A2–P2) interface. All imaging studies were reviewed by two independent echocardiographers blinded to the surgical technique.

Mitral regurgitation was graded as follows:

– 0 = none or trivial
– 1+ = mild
– 2+ = moderate
– 3+ = moderate to severe
– 4+ = severe

Only grades ≥2+ were considered clinically significant for the purpose of outcome assessment.

### Clinical follow-up and endpoints

All patients were followed up at discharge, 6 months, 1 year, and annually for at least 3 years and up to a maximum of 5 years. Follow-up evaluations included NYHA functional class assessment, clinical examination, TTE, and review of adverse events including mortality, endocarditis, stroke, and need for mitral valve reintervention.

Primary endpoint:

– Freedom from moderate or severe MR (≥2+) at 5 years

Secondary endpoints:

– NYHA class I–II at 5 years
– Freedom from reintervention
– Coaptation length ≥10 mm
– All-cause mortality

We selected ≥10 mm coaptation length as a pre-specified cut-off because it reflects our institutional quality target and is consistently described in prior surgical/echocardiographic literature as a “safety” value to secure a broad coaptation reserve and lower the risk of SAM. In other words, ≥10 mm is a conservative benchmark of technical success—not a claim that values <10 mm are failures—and aligns with previously reported practice standards.

### Statistical analysis

Statistical analysis was performed using SPSS version 27 (IBM Corp., Armonk, NY, USA).

We have assessed the distribution of continuous variables using the Shapiro–Wilk test, the results confirmed that all continuous variables were normally distributed, so continuous variables were reported as mean ± standard deviation and compared using unpaired Student's *t*-test. Categorical variables were expressed as counts and percentages and compared using chi-square or Fisher's exact test where appropriate. A *p*-value < 0.05 was considered statistically significant.

Kaplan–Meier survival analysis was used to estimate freedom from moderate/severe MR and freedom from reintervention. Interobserver variability in coaptation length measurement was assessed using intraclass correlation coefficients (ICCs).

## Results

Baseline characteristics were comparable between the two groups (Table 1). The mean patient age was 72.5 ± 4.2 years in the track group and 71.8 ± 3.9 years in the conventional group. All patients presented with isolated anterior leaflet prolapse or flail and underwent surgery through right mini-thoracotomy access in the majority of cases (80% track vs. 77% conventional, *p* = 0.81). No patients required conversion to valve replacement, and all received a complete annuloplasty ring without any additional surgical techniques.

Intraoperatively, the number of chordae implanted was similar between groups (2.1 ± 0.4 vs. 2.2 ± 0.5, *p* = 0.61). Aortic cross-clamp and cardiopulmonary bypass times did not differ significantly, indicating procedural feasibility of the track technique (Table 2).

**Table 2:** Intraoperative variables for both surgical techniques.

Variable	Track group	Conventional group	*p*-value
Aortic cross-clamp time (min) ± SD	52 ± 11	54 ± 13	0.54
Cardiopulmonary bypass time (min) ± SD	78 ± 15	81 ± 17	0.49
Mean number of chordae implanted ± SD	2.1 ± 0.4	2.2 ± 0.5	0.61
Complete annuloplasty (%)	100%	100%	—
Ring size	34 (32–38)	34 (32–38)	—
Conversion to valve replacement (%)	0%	0%	—

Postoperative outcomes were excellent in both groups, with zero 30-day mortality or major complications such as stroke or wound infections (Table 3). Length of hospital stay averaged 7.2 ± 1.5 days in the track group and 7.5 ± 1.8 days in the conventional group (*p* = 0.48).

**Table 3:** Early postoperative outcomes.

Variable	Track group	Conventional group	*p*-value
30-day mortality (%)	0%	0%	—
Neurological complications (%)	0%	0%	—
Wound infection (%)	0%	0%	—
Length of hospital stay (days) ± SD	7.2 ± 1.5	7.5 ± 1.8	0.48
Residual MR ≥ moderate (%)	0%	0%	—

**Figure 2:**
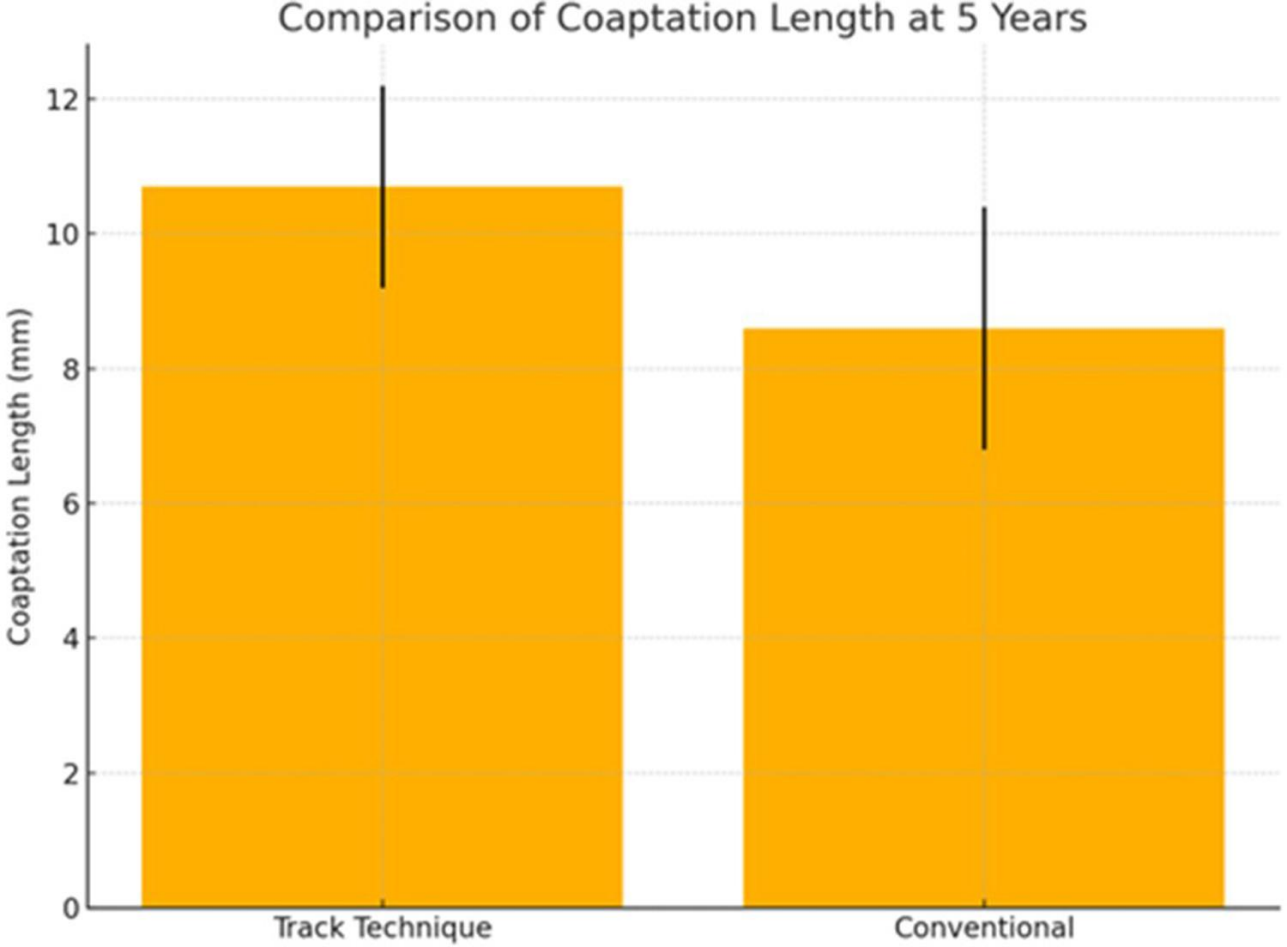
Comparison of coaptation length at 5-year follow-up between the track technique and the conventional approach.

At the 5-year follow-up mark, clinical and echocardiographic assessments were available for 85% of patients (40/47). No patient experienced mitral valve reoperation or developed endocarditis. NYHA class I–II was observed in 96% of patients in the track group and 91% in the conventional group (*p* = 0.52). Importantly, **residual MR of grade 1+** was significantly less common in the track group (12%) compared to the conventional group (32%, *p* = 0.04), suggesting better leaflet coaptation. Mean coaptation length, measured via intraoperative or early postoperative transesophageal echocardiography, was 10.7 ± 1.5 mm in the track group vs. 8.6 ± 1.8 mm in the conventional group (*p* = 0.03) (Table 4; Figure 2).

**Table 4:** Five-year follow-up clinical and echocardiographic outcomes.

Variable	Track group	Conventional group	*p*-value
All-cause mortality (%)	0% (0 patients)	4.5% (1 patient)	0.94
Mitral valve reoperation (%)	0%	0%	—
Residual MR ≥ moderate (%)	0%	0%	—
Residual MR grade 1+ (%)	12% (3 patients)	32% (7 patients)	0.04
NYHA class I–II (%)	96%	91%	0.52
Coaptation length (mm) ± SD	10.7 ± 1.5	8.6 ± 1.8	0.03

Further subgroup analysis revealed that patients in the track group showed not only greater coaptation length but also a narrower range of coaptation values (standard deviation: 1.5 mm vs. 1.8 mm), indicating reduced variability in repair outcomes. The mean number of implanted chordae did not differ significantly between groups. No patient in the track group exhibited signs of chordal entanglement or systolic anterior motion (SAM), while two patients in the conventional group required intraoperative adjustment due to suboptimal leaflet motion.

Postoperative left ventricular function, as assessed by echocardiographic ejection fraction, remained stable across follow-up in both groups, indicating that neither technique adversely affected ventricular performance.

## Discussion

Our 5-year comparative analysis confirms the central role of accurate chordal length determination in achieving durable mitral valve repair (MVr), particularly in the challenging setting of anterior leaflet prolapse or flail. While both the track and conventional techniques yielded excellent safety profiles and freedom from moderate or severe mitral regurgitation (MR), the track technique demonstrated superior echocardiographic precision, reflected by a significantly longer and more consistent coaptation length.

Coaptation length is a validated surrogate marker of repair durability (17). Insufficient leaflet coaptation is associated with early MR recurrence and suboptimal leaflet competence, particularly in anterior leaflet disease. Our findings reinforce this link: patients treated with the track technique achieved a mean coaptation length of 10.7 ± 1.5 mm, exceeding that of the conventional group and aligning with biomechanical thresholds previously associated with long-term competence. Furthermore, the narrower standard deviation in the track group (1.5 mm vs. 1.8 mm) indicates enhanced reproducibility and reduced inter-operator variability—critical goals in modern cardiac surgery. This consistency may translate into better long-term durability and fewer late reinterventions, although longer follow-up is needed.

While the mean number of implanted chordae did not differ significantly between groups, qualitative assessment suggested that the track technique facilitated more symmetric chordal tensioning and leaflet alignment. No patient in the track group exhibited signs of chordal entanglement or systolic anterior motion (SAM), while two patients in the conventional group required intraoperative adjustment due to suboptimal leaflet motion.

Postoperative left ventricular function, as assessed by echocardiographic ejection fraction, remained stable across follow-up in both groups, indicating that neither technique adversely affected ventricular performance. However, the improved coaptation seen in the track group may support more physiological leaflet motion and mitral competence over time.

Several strategies have been proposed to standardize neochordae length, including pre-measured loops (e.g., Chord-X), loop-based techniques (Leipzig loop/loop-in-loop), and template/caliper-guided sizing; robot-assisted measured-tube systems have also been described. While effective, these approaches may rely on preformed loop sizes, auxiliary instrumentation, or robotic platforms and therefore can have limited granularity or availability. In contrast, the track technique uses the annular plane after complete annuloplasty as a fixed reference, does not require ventricular pressurization, and is platform-agnostic (mini-thoracotomy or sternotomy). In our series, this translated into longer and more consistent coaptation compared with conventional visual estimation.

Recent studies have emphasized the importance of standardized techniques to achieve consistency in MVr outcomes. Tomsi et al. (18) reported that standardized procedural pathways significantly improved postoperative recovery and reduced adverse events, especially in elderly patients undergoing minimally invasive MVr. Our findings suggest that the track technique, by eliminating subjectivity and anchoring chordal length to an anatomical landmark, aligns well with such structured approaches and may help extend high-quality repair outcomes beyond high-volume centers.

While chordal replacement with expanded polytetrafluoroethylene (ePTFE) has become well-established, recent long-term data reinforce its durability when precise length control is achieved. McCarthy et al. (19) demonstrated low rates of MR recurrence and excellent valve function beyond 8 years post-repair, underlining the value of reproducible chordal implantation. These outcomes mirror our own and support the premise that repair success hinges not only on materials used, but on geometric precision during implantation.

To this end, innovations in robotic and device-assisted chordal sizing have emerged. Tedoriya et al. (14) introduced the “measured tube technique”, using a calibrated delivery system during robotic MVr to predefine chordal length. While technologically sophisticated, such systems require robotic platforms and specific instrumentation. In contrast, the track technique provides similar accuracy without dependence on robotics, making it more accessible to a broad range of surgical teams.

Emerging technologies such as augmented reality (AR) and 3D intraoperative imaging are also shaping the future of MVr. Moon et al. (15) demonstrated that AR enhances surgical planning and anatomic orientation, potentially aiding complex repairs. While not yet integrated into routine practice, such tools could complement the track technique by providing enhanced visual feedback and augmenting intraoperative confidence, particularly in minimally invasive or robotic environments.

From a biomechanical perspective, optimal chordal length achieves symmetric leaflet motion, reduces chordal stress, and maximizes coaptation. Rin et al. (16) used finite element modeling to show that even submillimetric discrepancies in chordal length disrupt stress distributions and coaptation geometry. This computational evidence provides mechanistic validation for the track technique, which leverages a fixed annular reference to achieve consistent geometrical alignment across diverse anatomies.

Beyond clinical outcomes, the track technique has educational value. Its intuitive, pressurization-independent methodology offers a reproducible learning curve for junior surgeons. Unlike visual or saline-based estimation, it minimizes subjective interpretation, fostering surgical confidence and skill acquisition. As the demand for MVr grows in less experienced centers, the dissemination of simple yet effective techniques becomes critical for ensuring repair quality and patient safety.

In summary, this 5-year follow-up study confirms that the track technique not only matches conventional methods in safety but also provides superior coaptation geometry, reduced variability, and long-term MR suppression. The integration of recent evidence—from device innovations and AR to computational biomechanics—reinforces the importance of precision in neo-chordal implantation. The track technique, with its simplicity, anatomical logic, and reproducibility, may serve as a benchmark for standardized, high-quality mitral repair across varied surgical platforms.

### Limitations and future perspectives

Despite its strengths, this study has several limitations that warrant careful consideration. First, its retrospective design introduces potential selection and observational biases, despite rigorous inclusion criteria and uniform surgical protocols. Randomized controlled trials remain the gold standard for comparing surgical interventions, and future prospective trials are needed to confirm the superiority of the track technique over alternative methods.

Second, all procedures were conducted at a single high-volume mitral center by experienced surgeons. While this ensures a high level of procedural consistency, it limits generalizability. Outcomes in lower-volume institutions or among surgeons at different stages of the learning curve may differ, and additional multicenter studies are necessary to validate the technique across broader surgical environments.

Third, although the track technique appears to offer enhanced consistency in coaptation geometry and MR reduction, our study did not include advanced imaging modalities such as 3D transesophageal echocardiography or intraoperative strain analysis to quantify leaflet motion and chordal tension. Incorporating such tools in future studies could provide deeper insight into the mechanical advantages of annular-guided chordal implantation.

Furthermore, we did not assess patient-reported outcomes (e.g., quality of life, functional capacity) or long-term leaflet stress patterns that may correlate with repair durability. These variables are becoming increasingly important in the era of patient-centered care and should be included in future prospective designs.

Future directions for research should include the integration of the track technique with advanced intraoperative imaging and navigational tools, such as augmented reality, to improve spatial orientation and real-time chordal placement accuracy. Additionally, the development of modular guiding devices tailored to robotic and mini-thoracotomy access routes could enhance the adaptability of the technique in diverse surgical settings.

The biomechanical principles underpinning the track technique should also be explored through computational modeling, cadaveric studies, and ex vivo simulation to optimize design parameters and understand the interaction between annular geometry, chordal tension, and leaflet dynamics. Such investigations may help refine the technique further and tailor it to individual anatomical variations.

Lastly, the role of the track technique in the context of valve repair complexity should be evaluated. While it appears especially beneficial in isolated anterior leaflet prolapse, its application to bileaflet pathology or functional MR remains unexplored and may represent a future area of innovation.

In conclusion, while the current study supports the safety, effectiveness, and reproducibility of the track technique, continued refinement, validation, and integration with emerging surgical technologies will be essential to fully realize its potential as a transformative tool in mitral valve repair.

## Conclusion

In this 5-year analysis of mitral valve repair (MVr) for anterior leaflet prolapse or flail, the track technique showed excellent durability and superior echocardiographic outcomes compared to conventional chordal length estimation. It resulted in longer, more consistent coaptation and less residual mitral regurgitation.

The technique offers procedural advantages by eliminating the need for intraoperative pressurization or subjective assessments, making it especially valuable in minimally invasive and robotic settings. Its standardization enhances reproducibility, supports training in lower-volume centers, and reduces operator dependence.

By providing objective anatomical guidance, the track technique facilitates skill acquisition and may contribute to more uniform surgical quality. As mitral repair trends toward minimally invasive and precise methods, this technique aligns with future demands for scalable, reliable solutions.

## Data Availability

The original contributions presented in the study are included in the article/Supplementary Material, further inquiries can be directed to the corresponding author.
